# Book Reviews

**Published:** 1899-03

**Authors:** 


Transactions of the State Medical Society of Wisconsin for the Year 1898. Vol. XXXII: Edited by Charles S. Sheldon, M. D., Secretary. Octavo, pp. xiv.—583. Madison, Wis.: Tracy, Gibbs & Co., Printers. 1898.

This excellent state society again presents its yearly work for inspection. It is a record that will bear the closest scrutiny and sustain the severest test of criticism. After the preliminary matter, which contains the proceedings of the meeting and the president's address, the work is classified into materia medica and therapeutics, surgery, practice of medicine, neurology, gynecology, obstetrics, pediatrics, ophthalmology, otology and laryngology, state medicine and hygiene, and a medico-legal section. Under each of these heads several interesting papers are published. This society, like many others, is struggling with the question of an excess of material offered and how to deal with it. It has instructed its program committee hereafter to accept not to exceed fifty papers—one half to be read by invitation, and one half by volunteers. We shall observe with interest the practical working of this plan.

---

Transactions of the Ohio State Medical Society at its Fifty-third Annual Meeting, held at Columbus, May 4, 5 and 6, 1898. Edited by Dr, P. Maxwell Foshay. Small 8vo, pp. 558. Cleveland, O.: J. B. Savage. Press. 1898.

This volume is well arranged and printed with care, showing the supervisory work of an experienced secretary. A most excellent portrait of Dr. W. H. Humiston, the president, is published as a frontispiece. The state board of medical registration and examination, through its president, Dr. N. R. Coleman, of Columbus, presented an interesting report, which is a summary of the work of the previous year. From it we note that the board instituted eighty-two prosecutions during the year for violations of the law. Of these twenty-three were convicted, seven acquitted, five failed of indictment by the grand jury, and forty-nine were pending. The influence of such work is excellent, and we look forward hopefully to the time when similar prosecutions may be instituted in New York. At present we are very much handicapped in this respect, our law being defective on this point.

The volume presents a number of interesting papers, duly classified in regard to the several branches of medicine. The society did itself credit in selecting Dr. N. R. Coleman as president for the coming year. The next meeting will be held at Springfield, May 10, 11 and 12, 1899.

TWENTIETH CENTURY PRACTICE. An International Encyclopedia of Modern Medical Science. By leading authorities of Europe and America. Edited by THOMAS L. STEDMAN, M. D., New York City. In twenty volumes. Volume XVII. Infectious Diseases and Malignant Neoplasms. New York: William Wood & Co. 1898.

The consideration of infectious diseases is completed in this volume and malignant neoplasms are added to finish out the book. The publishers apologise for the appearance of this volume out of its natural order—*i. e.*, before Volume XVI., but we see no harm in this, for practically each book is independent of the other in dealing with the several diseases.

In this number the general pathology and bacteriology of diphtheria are treated of by Dr. William Hallock Park, of New York, and its symptomatology and treatment is dealt with by Dr. A. Jacobi, of the same city. Taken together, these dissertations constitute the most important text-book literature on the subject that has yet appeared. The next subject considered is tetanus, and this is somewhat exhaustively handled by Professor Victor Baber, of Bucharest. He regards the treating of this disease by antitoxin as a distinct advance, but does not advise the omission of other means. He speaks from an experience of seven cases treated with antitoxin.

By far the largest part of the book is taken up with the exploitation of cancer, its general pathology, being described by Mr. W. Roger Williams, of Bristol, Eng., and its symptomatology and treatment by Dr. William B. Cooley, of New York. The study of this disease possesses an added interest in view of the researches going on at the state laboratory in this city, under the direction of Dr. Roswell Park, assisted by Dr. H. R. Gaylord. It is one of the most important questions that can engage the attention of the profession, and we shall contemplate with interest any contribution that may throw light upon it. The articles in this volume treat of it intelligently in the light of our present knowledge—we mean published knowledge. The general pathology of sarcoma is also considered by Roger Williams, and its symptomatology and treatment by Dr. Cooley.

The malignant new growths of the skin form a section which is written by Dr. John T. Bowen, of Boston, and malignant diseases of the female organs of generation, by Dr. Edward McGuire, of Richmond, Va., closes this interesting volume. Dr. McGuire is especially interesting, as well as instructive in dealing with his subject, and what he says is a distinct contribution to its literature. This volume more than keeps pace with some of its predecessors in interest and importance.

---

Annual and Analytical Cyclopedia of Practical Medicine. By Charles E. de M. Sajous, M. D., and 100 associate editors. Assisted by corresponding editors, collaborators and correspondents. Illustrated with chromo-lithographs, engravings and maps. Volume II. Bromide of Ethyl to Diphtheria. Philadelphia, New York and Chicago: F. A. Davis Co. 1899.

The change in the form of the annual has proven very satisfactory. The first volume was, of course, something of an experiment, but now the work is past the experimental stage and its standing is assured. This volume inaugurates the regular plan of the work, wherein all articles have been prepared by their respective editors. In the first the majority of the articles were immediately supervised by the editor-in-chief. Dr. Sajous discloses the fact in the preface to this volume, that the unsigned article on "Animal Extracts," in the first volume was written by himself—a bit of information that readers will be glad to learn, since it was one of the most complete and scientific expositions of the subject that has yet appeared in print.

A number of articles of exceptional value on very important subjects are published in this volume, and the editor himself very properly invites special attention to them. Among them are: Cerebral hemorrhage, by Dr. William Browning, of Brooklyn; Cirrhosis of the liver, by Professor Adami, of Montreal; Diphtheria, by Drs. Northrup and Bovaird, of New York; Cocainomania, by Dr. Norman Kerr, of London, and Dilatation of the heart, by Dr. Vickery, of Boston. There are others we should like to mention, but our space is limited, and it must suffice for us to say that every one who desires to keep pace with the literature of medicine will get the book and examine it for himself.

---

A Pocket Medical Dictionary, Giving the Pronunciation and Definition of the Principal Words Used in Medicine and the Collateral Sciences. By George M. Gould, A. M., M. D., author of The Illustrated Medical Dictionary, The Student's Medical Dictionary; Editor of the Philadelphia Medical Journal, etc. New edition, rewritten and enlarged, including over 21,000 words. Philadelphia: P. Blakiston's Son & Co. 1898.

A good pocket dictionary is desirable for student and physician. The former needs to keep it near by, even in the lecture room, for frequent consultation, and the latter always needs it in his bed-room, where he may refer to it at such times as may prove inconvenient for him to visit his library. This book, now in its second edition, meets all the requirements of a pocket dictionary. It has been rewritten and enlarged, bringing it forward to the requirements of the period. It contains all the information necessary for an emergency, and this after all is the proper function and is all that is demanded of such a lexicon. There is none better in the field.

---

The American Year-Book of Medicine and Surgery. Being a yearly digest of scientific progress and authoritative opinion in all branches of medicine and surgery, drawn from journals, monographs and text-books of the leading American and foreign authors and investigators. Collected and arranged with critical editorial comments by eminent American specialists and teachers. Under the general editorial charge of George M. Gould, M. D. Illustrated. Philadelphia: W. B. Saunders. 1899.

The editor of this annual and his assistants have again collected, digested, and arranged an enormous quantity of material for the use of the practitioner of medicine. It is well to have the literature of the previous year made so readily available as it is in this book and our special wonder is how it can be done so quickly and well, and, withal, so cheaply. The price of this book—$6.50, in cloth—is a mere bagatelle compared with its real value to a busy physician. Its possession should by no means prevent him from taking a reasonable number of medical journals, but it will save him the necessity of purchasing a number of almost useless books that are offered in one way and another for his money and which he is oftentimes persuaded to purchase against his best judgment.

This volume follows in the pathway of its predecessors, and is constructed on quite similar lines. It makes a creditable showing for all in any manner connected with it.

---

PRACTICAL URANALYSIS AND URINARY DIAGNOSIS: A Manual for the Use of Physicians, Surgeons and Students. By CHARLES W. PURDY, M. D., LL. D. (Queen's University); Fellow of the Royal College of Physicians and Surgeons, Kingston; Professor of Clinical Medicine at the Chicago Post-Graduate Medical School. Fourth, revised edition. Crown 8vo, pp. 365. Copiously illustrated. Philadelphia: The F. A. Davis Co. 1898.

This excellent manual will be welcomed in its fourth edition by all its many old friends, and we trust a goodly proportion of new ones will make haste to give it a cordial greeting. As it has been adopted as a text-book in sixty or more medical colleges, and as its three earlier editions were exhausted within as many years, and, moreover, because the profession in general has given it a most favorable reception, three very cogent reasons are thus furnished for the author to continue in his good work. He has made extended changes in the department relating to the chemistry of the urine, some of the chapters having been entirely rewritten. A number of new illustrations have been added and the whole subject has been brought forward to the immediate present.

